# Association Between Visceral Fat and Brain Cortical Thickness in the Elderly: A Neuroimaging Study

**DOI:** 10.3389/fnagi.2021.694629

**Published:** 2021-06-23

**Authors:** Jaelim Cho, Seongho Seo, Woo-Ram Kim, Changsoo Kim, Young Noh

**Affiliations:** ^1^Institute for Environmental Research, Yonsei University College of Medicine, Seoul, South Korea; ^2^Institute of Human Complexity and Systems Science, Yonsei University, Incheon, South Korea; ^3^Department of Electronic Engineering, Pai Chai University, Daejeon, South Korea; ^4^Department of Neuroscience, College of Medicine, Gachon University, Incheon, South Korea; ^5^Neuroscience Research Institute, Gachon University, Incheon, South Korea; ^6^Department of Preventive Medicine, Yonsei University College of Medicine, Seoul, South Korea; ^7^Department of Neurology, Gil Medical Center, Gachon University College of Medicine, Incheon, South Korea; ^8^Department of Health Science and Technology, GAIHST, Gachon University, Incheon, South Korea

**Keywords:** abdominal fat, visceral fat, neuroimaging, cortical thickness, MRI

## Abstract

**Background:**

Despite emerging evidence suggesting that visceral fat may play a major role in obesity-induced neurodegeneration, little evidence exists on the association between visceral fat and brain cortical thickness in the elderly.

**Purpose:**

We aimed to examine the association between abdominal fat and brain cortical thickness in a Korean elderly population.

**Methods:**

This cross-sectional study included elderly individuals without dementia (*n* = 316). Areas of visceral fat and subcutaneous fat (cm^2^) were estimated from computed tomography scans. Regional cortical thicknesses (mm) were obtained by analyzing brain magnetic resonance images. Given the inverted U-shaped relationship between visceral fat area and global cortical thickness (examined using a generalized additive model), visceral fat area was categorized into quintiles, with the middle quintile being the reference group. A generalized linear model was built to explore brain regions associated with visceral fat. The same approach was used for subcutaneous fat.

**Results:**

The mean (standard deviation) age was 67.6 (5.0) years. The highest quintile (vs. the middle quintile) group of visceral fat area had reduced cortical thicknesses in the global [β = –0.04 mm, standard error (SE) = 0.02 mm, *p* = 0.004], parietal (β = –0.04 mm, *SE* = 0.02 mm, *p* = 0.01), temporal (β = –0.05 mm, *SE* = 0.02 mm, *p* = 0.002), cingulate (β = –0.06 mm, *SE* = 0.02 mm, *p* = 0.01), and insula lobes (β = –0.06 mm, *SE* = 0.03 mm, *p* = 0.02). None of the regional cortical thicknesses significantly differed between the highest and the middle quintile groups of subcutaneous fat area.

**Conclusion:**

The findings suggest that a high level of visceral fat, but not subcutaneous fat, is associated with a reduced cortical thickness in the elderly.

## Introduction

Obesity is a well-known risk factor for cardiovascular diseases, type 2 diabetes, and cancer (Bogers et al., 2007; Renehan et al., 2008; Bell et al., 2014). It has also been suggested that obesity is an independent risk factor for Alzheimer’s disease and vascular dementia (Beydoun et al., 2008). To elucidate the effect of obesity on the brain in cognitively healthy individuals, a number of neuroimaging studies have investigated the association between obesity (including central obesity) and brain structure on magnetic resonance imaging (MRI) (Gunstad et al., 2008; Taki et al., 2008; Raji et al., 2010; Yokum et al., 2012; Kurth et al., 2013; Kim et al., 2015; Medic et al., 2016; Dekkers et al., 2019; Hamer and Batty, 2019; Morys et al., 2021). A large-scale study of the United Kingdom Biobank (*n* = 9,652) showed that three obesity indices [body mass index (BMI), waist-to-hip ratio (WHR), and total fat mass from body impedance] were significantly associated with a reduction in global gray matter volume (Hamer and Batty, 2019). Another study of the United Kingdom Biobank (*n* = 12,087) reported that the association between total fat mass from body impedance and global gray matter volume was significant only in men (Dekkers et al., 2019). Some of the neuroimaging studies have measured cortical thickness (Kim et al., 2015; Medic et al., 2016; Morys et al., 2021), a more sensitive indicator of gray matter changes than cortical volume (Burggren et al., 2008; Thambisetty et al., 2010). Kim et al. (2015) demonstrated inverse associations between total fat percentage from body impedance and WHR with region-of-interest (ROI)-based global and frontal thicknesses only in men. Medic et al. (2016) found several focal regions in the frontal and occipital lobes inversely associated with BMI6). Morys et al. (2021) reported that BMI, WHR, and body fat percentage were associated with thinner temporal, entorhinal, orbitofrontal, and cingulate cortices, as well as thicker frontal, parietal, and occipital cortices.

Emerging neuroimaging studies have suggested the role of visceral fat in the association between obesity and brain structures in adults (Debette et al., 2010; Isaac et al., 2011; Widya et al., 2015; Zsido et al., 2019). Debette et al. (2010) demonstrated that visceral fat on computed tomography (CT) had the strongest association with reduced total brain volumes when compared with other obesity indices (BMI, waist circumference, wait-to-hip ratio, and subcutaneous fat), and the association was independent of BMI and insulin resistance. Widya et al. (2015) reported that increased visceral fat (but not subcutaneous fat) was associated with microstructural brain tissue damage in the elderly. Zsido et al. (2019) demonstrated that increased visceral fat was associated with accelerated brain aging (based on structural brain networks derived from gray matter volume, cortical thickness, and surface area) in adults including elderly participants. Isaac et al. (2011) analyzed the data of 184 healthy elderly individuals using voxel-based morphometry, and found that visceral fat was inversely associated with cortical thicknesses in several focal regions (e.g., pre-central, post-central, superior temporal, and inferior parietal cortices). Although the ROI-based approach (compared with voxel-based morphometry) can facilitate clinical interpretation by predefining brain regions, no study has investigated the associations between visceral fat (as well as subcutaneous fat) and ROI-based cortical thicknesses.

Hence, the present study aimed to explore brain regions associated with abdominal fat in the elderly, using the ROI-based analysis of brain magnetic resonance images.

## Materials and Methods

### Study Participants

This study recruited ≥ 60 year-old individuals (without self-reported history of dementia, movement disorders, or stroke) through local advertisements between December 2015 and September 2017 in Incheon, Republic of Korea, as part of the EPINEF study. The survey was conducted at Gachon University Gil Medical Center (Incheon, South Korea). Using a standardized survey protocol, a total of 322 participants completed questionnaires (regarding demographic characteristics, medical history, and lifestyle behaviors), anthropometric measurement (weight and height), blood sampling, abdominal fat CT scans, mini-mental state examination (MMSE), and brain 3T MRI scans. Two participants who were found to have brain tumors on brain MRI were excluded. After excluding individuals with missing values, 316 participants (129 men and 187 women) were included in the study. All individuals provided written informed consent. The study was approved by the Institutional Review Board of Gachon University Gil Medical Center (approval No. GDIRB2015-225).

### Acquisition of Abdominal Fat Areas

All subjects underwent 10-mm-slice CT scans (SOMATOM Sensation 64; Siemens Healthcare, Forchheim, Germany) at the umbilical level. The average value of pixels within the range of –200 to –20 Hounsfield units was used for the measurement of abdominal fat areas (Jackson and Thomas, 2004). The total visceral fat area and the subcutaneous fat area (unit: cm^2^) were measured with a commercial software program (syngo Volume; Siemens Healthcare, Forchheim, Germany).

### Acquisition of Brain Imaging Markers

Brain 3D-T1-magnetization-prepared rapid gradient-echo (MP-RAGE) images were obtained with a Siemens 3T Verio MRI, using a standardized MRI protocol. The image parameters used for 3D T1-MP-RAGE were as follows: repetition time, 1,900 ms; echo time, 2.93 ms; flip angle, 8°; pixel bandwidth, 170 Hz/pixel; matrix size, 256 × 208; field of view, 256 mm; number of excitations, 1; total acquisition time, 4 min 10 s; voxel size, 1.0 × 1.0 × 1.0 mm^3^.

ROI-based analyses of the brain images were performed using the standard FreeSurfer 6.0.0 pipeline^1^, which consists of subcortical segmentation (Fischl et al., 2002, 2004a); cortical surface reconstruction (Dale et al., 1999; Fischl et al., 1999); cortical thickness mapping (Fischl and Dale, 2000); surface-based inter-subject alignment (Fischl et al., 1999); and cortical parcellation (Fischl et al., 2004b; Desikan et al., 2006). Using these serial procedures, we obtained estimates of regional cortical thickness (frontal, temporal, parietal, occipital, cingulate, and insula) and subcortical gray matter volume (thalamus, caudate, putamen, pallidum, hippocampus, amygdala, and nucleus accumbens). Global cortical thickness was calculated by averaging the six cortical thicknesses.

### Covariates

The questionnaire included educational years, history of disease (hypertension, diabetes mellitus, dyslipidemia, and angina or myocardial infarction), smoking status (never, former, or current smoker), and alcohol consumption (currently drinking or not). Measured weight and height were used to calculate BMI (unit: kg/m^2^). At least 12-h fasting blood samples were tested for blood glucose and total cholesterol levels, and apolipoprotein E (APOE) genotyping.

### Statistical Analysis

**FIGURE 1:**
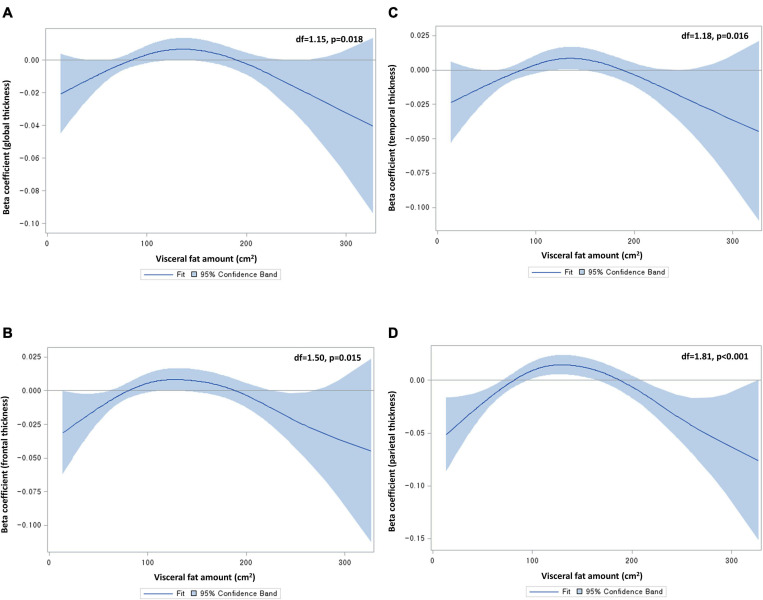
Non-linear relationships of visceral fat area with **(A)** global, **(B)** frontal, **(C)** temporal, and **(D)** parietal cortical thicknesses. Df, degrees of freedom. Beta coefficients were from generalized additive models, adjusting for age, sex, educational years, hypertension, diabetes, dyslipidemia, angina or myocardial infarction, smoking status, alcohol consumption, apolipoprotein status, body mass index, fasting blood glucose level, total cholesterol level, and intracranial volume. Degrees of freedom were determined by the cross-validation method.

To explore the non-linear relationship between abdominal fat area and cortical thickness, we used a generalized additive model (GAM), including visceral fat area as a spline variable and global cortical thickness as a dependent variable. In this analysis, we adjusted for age, sex, educational years, hypertension, diabetes, dyslipidemia, angina or myocardial infarction, smoking status, alcohol consumption, APOE status (presence/absence of ε4 allele), BMI, fasting blood glucose level, total cholesterol level, and intracranial volume (ICV). The degrees of freedom for the spline variable were automatically selected using the generalized cross validation method. A two-sided *p* < 0.05 from analysis of deviance for GAM was considered as having a significant non-linear relationship. There were significant non-linear relationships of visceral fat area with global (*p* = 0.02), frontal (*p* = 0.02), temporal (*p* = 0.02), and parietal thicknesses (*p* < 0.001), with an inverted U shape (Figure 1). Hence, we classified visceral fat area into quintiles (quintile 5 as the highest; quintile 3 as the reference group) and entered the quintiles into a generalized linear model (GLM). This approach was used for all the regional cortical thicknesses (though occipital and insular thicknesses did not exhibit significant non-linear relationships) with a view to presenting results in a consistent manner. The same method was applied to subcutaneous fat area (albeit none of the non-linear relationships were significant) in order to enable a straightforward comparison with visceral fat. Given the absence of a significant non-linear relationship in the GAM analysis of subcortical volumes, the abdominal fat variables were entered as a continuous variable into the GLM for subcortical volumes. All GLM analyses were conducted after adjusting for the same covariates as the above GAM. Given possible sex differences in abdominal fat distribution as well as brain MRI markers (e.g., cortical thickness and volume) (Ritchie et al., 2018), sex-stratified analyses were additionally conducted. Significance of sex differences was tested using the method described by Altman and Bland and expressed as p for interaction (Altman and Bland, 2003). All analyses were corrected for multiple comparisons using the false discovery rate (FDR) method (Benjamini and Hochberg, 1995).

A *post hoc* analysis was conducted to examine the associations of other obesity indices (BMI and waist circumference) with brain cortical thickness. Quintiles of either BMI or waist circumference were entered into GLMs, with adjustment for the same covariates as the main analysis.

All statistical analyses were conducted using SAS version 9.4 (SAS Institute, Cary, NC, United States). Two-sided *p* < 0.05 were considered statistically significant.

## Results

### Characteristics of Study Participants

The mean [standard deviation (SD)] age of the study participants was 67.6 (5.0) (Table 1). The numbers of those with hypertension and dyslipidemia were 134 (42.4%) and 104 (32.9%), respectively. The mean (SD) areas of visceral fat and subcutaneous fat were 125.3 (55.4) cm^2^ and 167.5 (65.7) cm^2^, respectively. The mean (SD) MMSE score was 28.3 (1.9). The mean (SD) global thickness was 2.5 (0.1) mm.

**TABLE 1:** Characteristics of the study participants.

	**Total (*N* = 316)**	**Men (*N* = 129)**	**Women (*N* = 187)**
Age, mean (SD)	67.6 (5.0)	68.9 (4.9)	66.7 (5.0)
Educational years, mean (SD)	9.6 (4.3)	11.1 (4.1)	8.6 (4.0)
Hypertension, N (%)	134 (42.4)	60 (46.5)	74 (39.6)
Diabetes mellitus, N (%)	62 (19.6)	32 (24.8)	30 (16.0)
Dyslipidemia, N (%)	104 (32.9)	35 (27.1)	69 (36.9)
Angina or myocardial infarction, N (%)	37 (11.7)	19 (14.7)	18 (9.6)
**Smoking status, N (%)**			
Never smoker	214 (67.7)	29 (22.5)	185 (98.9)
Former smoker	79 (25.0)	77 (59.7)	2 (1.1)
Current smoker	23 (7.3)	23 (17.8)	0 (0.0)
Alcohol drinking, N (%)	113 (35.8)	75 (58.1)	38 (20.3)
Body mass index, mean (SD)	24.7 (3.1)	24.8 (2.6)	24.7 (3.4)
**Apolipoprotein status, N (%)**			
At least one ε4 allele	56 (17.7)	26 (20.2)	30 (16.0)
No ε4 allele	260 (82.3)	103 (79.8)	157 (84.0)
Fasting blood glucose, mean (SD)	99.0 (21.7)	100.0 (22.3)	98.4 (21.4)
Total cholesterol, mean (SD)	183.7 (37.7)	176.1 (38.0)	189.0 (36.7)
**Abdominal fat (cm^2^), mean (SD)**			
Visceral fat	125.3 (55.4)	135.9 (59.9)	118.0 (51.0)
Subcutaneous fat	167.5 (65.7)	130.1 (46.8)	193.3 (64.5)
ICV (mm^3^), mean (SD)	1,251,894 (126,138)	1,346,200 (101,857)	1,186,839 (96,866)
**Cortical thickness (mm), mean (SD)**			
Global	2.5 (0.1)	2.5 (0.1)	2.5 (0.1)
Frontal lobe	2.5 (0.1)	2.5 (0.1)	2.6 (0.1)
Parietal lobe	2.8 (0.1)	2.7 (0.1)	2.8 (0.1)
Temporal lobe	2.3 (0.1)	2.2 (0.1)	2.3 (0.1)
Occipital lobe	2.0 (0.1)	2.0 (0.1)	2.0 (0.1)
Cingulate	2.6 (0.1)	2.5 (0.1)	2.6 (0.1)
Insula	2.9 (0.1)	2.9 (0.1)	2.9 (0.1)
**Subcortical volume (mm^3^), mean (SD)**			
Thalamus	6474.9 (649.2)	6691.9 (666.2)	6325.2 (594.2)
Caudate	3256.3 (477.7)	3444.6 (476.3)	3126.4 (434.5)
Putamen	4461.1 (520.1)	4651.5 (534.4)	4329.7 (468.0)
Pallidum	1894.8 (205.4)	1960.8 (195.8)	1849.3 (199.8)
Amygdala	1637.6 (193.7)	1707.5 (187.4)	1589.4 (183.5)
Hippocampus	3903.3 (379.9)	3993.8 (363.4)	3840.9 (379.3)
Nucleus accumbens	427.4 (75.0)	447.1 (78.8)	413.8 (69.3)

*SD, standard deviation, ICV, intracranial volume.*

### Association Between Abdominal Fat Area and Cortical Thickness

The quintile 3 group of visceral fat area had the greatest global cortical thickness (mean, 2.52 mm; SD, 0.07 mm), whereas the quintile 5 group had the smallest (mean, 2.48 mm; SD, 0.08 mm) (Table 2). In the GLM analysis of visceral fat (Table 3), the quintile 5 group (vs. the quintile 3 group) had significantly reduced cortical thicknesses in the global [β = –0.04 mm, standard error (SE) = 0.02 mm, *p* = 0.004], parietal (β = –0.04 mm, *SE* = 0.02 mm, *p* = 0.01), temporal (β = –0.05 mm, *SE* = 0.02 mm, *p* = 0.002), cingulate (β = –0.06 mm, *SE* = 0.02 mm, *p* = 0.01), and insula lobes (β = –0.06 mm, *SE* = 0.03 mm, *p* = 0.02). These associations remained significant after FDR correction. In men, the quintile 5 group (vs. the quintile 3 group) had significantly reduced global, temporal, and insular thicknesses, though these associations did not remain significant after FDR correction. In women, the quintiles 2, 4, and 5 groups had significantly reduced global cortical thicknesses, as compared with the quintile 3 group. The quintile 5 group (vs. the quintile 3 group) among women also had a significantly reduced parietal thickness (β = –0.04 mm, *SE* = 0.02 mm, *p* = 0.04). After FDR correction, reduced global thicknesses in the quintiles 2 and 3 groups, a reduced frontal thickness in the quintile 4 group, a reduced parietal thickness in the quintile 1 group, a reduced occipital thickness in the quintile 1 group, and a reduced cingulate thickness in the quintile 2 group remained significant. Regarding sex differences, the quintile 2 (vs. quintile 3) group had reduced cortical thicknesses among women but increased thicknesses among men in the global (p for interaction = 0.024), frontal (p for interaction = 0.036), parietal (p for interaction = 0.007), and cingulate lobes (p for interaction = 0.021). Otherwise sex differences were not significant.

**TABLE 2:** Global cortical thickness by quintiles of abdominal fat area.

		**Abdominal fat area**				**Global cortical thickness**	
	**N**	**Mean**	***SD***	**Minimum**	**Maximum**	**Mean**	***SD***
**Visceral fat**							
**Total (*N* = 316)**							
Quintile 1	63	54.68	15.51	13.66	74.13	2.50	0.08
Quintile 2	63	91.12	9.48	74.58	107.92	2.50	0.08
Quintile 3	64	120.08	7.96	108.22	135.14	2.52	0.07
Quintile 4	63	153.20	10.40	135.50	171.18	2.51	0.08
Quintile 5	63	207.75	33.47	171.24	326.45	2.48	0.08
**Men (*N* = 129)**							
Quintile 1	25	53.24	18.36	13.66	75.27	2.47	0.08
Quintile 2	26	98.14	13.70	75.81	117.24	2.50	0.08
Quintile 3	26	134.38	10.32	118.03	150.33	2.50	0.08
Quintile 4	26	170.17	9.72	152.63	187.48	2.47	0.07
Quintile 5	26	220.63	25.49	191.04	280.17	2.46	0.07
**Women (*N* = 187)**							
Quintile 1	37	55.19	13.42	16.87	73.47	2.52	0.07
Quintile 2	38	87.99	8.26	73.71	99.87	2.50	0.09
Quintile 3	37	112.48	6.75	100.05	125.28	2.55	0.06
Quintile 4	38	140.85	10.77	125.62	158.11	2.51	0.07
Quintile 5	37	193.86	39.00	159.1	326.45	2.52	0.08
**Subcutaneous fat**							
**Total (*N* = 316)**							
Quintile 1	63	90.06	23.79	15.75	115.14	2.48	0.07
Quintile 2	63	127.14	7.41	115.51	140.91	2.49	0.09
Quintile 3	64	157.95	9.71	142.03	173.87	2.51	0.07
Quintile 4	63	194.44	13.05	174.85	220.18	2.50	0.07
Quintile 5	63	268.05	47.02	221.54	417.81	2.52	0.08
**Men (*N* = 129)**							
Quintile 1	25	69.99	21.65	15.75	92.57	2.46	0.07
Quintile 2	26	106.58	7.88	93.16	116.16	2.48	0.08
Quintile 3	26	125.29	4.80	116.23	133.09	2.47	0.10
Quintile 4	26	146.75	7.65	133.71	160.05	2.49	0.07
Quintile 5	26	199.35	33.93	163.84	306.01	2.48	0.06
**Women (*N* = 187)**							
Quintile 1	37	114.85	18.21	40.03	138.36	2.51	0.07
Quintile 2	38	155.22	9.93	139.16	170.92	2.52	0.07
Quintile 3	37	184.30	8.88	171.04	198.74	2.53	0.07
Quintile 4	38	222.01	12.63	199.73	242.21	2.51	0.08
Quintile 5	37	290.52	48.13	243.25	417.81	2.54	0.08

*SD, standard deviation.*

**TABLE 3:** Association between visceral fat area and cortical thickness.

		**Total (*N* = 316)**			**Men (*N* = 129)**			**Women (*N* = 187)**			***p* for interaction^†^**
		**Beta**	***SE***	***p***	**Beta**	***SE***	***p***	**Beta**	***SE***	***p***	
Global	Quintile 1 vs. 3	–0.008	0.015	0.61	–0.022	0.024	0.35	–0.022	0.02	0.27	1.00
	Quintile 2 vs. 3	–0.01	0.014	0.45	0.017	0.023	0.45	**–0.049**	**0.018**	0.009*	0.024
	Quintile 4 vs. 3	–0.011	0.014	0.42	–0.035	0.023	0.13	**–0.05**	**0.018**	0.007*	0.61
	Quintile 5 vs. 3	**–0.043**	**0.015**	0.004*	**–0.05**	**0.024**	**0.042**	**–0.04**	**0.019**	**0.038**	0.74
Frontal	Quintile 1 vs. 3	–0.019	0.017	0.27	–0.014	0.025	0.58	–0.035	0.022	0.12	0.53
	Quintile 2 vs. 3	–0.007	0.015	0.63	0.027	0.024	0.28	–0.04	0.021	0.055	0.036
	Quintile 4 vs. 3	–0.013	0.015	0.40	–0.019	0.024	0.44	**–0.059**	**0.021**	0.004*	0.21
	Quintile 5 vs. 3	–0.028	0.016	0.082	–0.014	0.026	0.58	–0.035	0.021	0.11	0.53
Parietal	Quintile 1 vs. 3	–0.031	0.017	0.069	–0.03	0.029	0.29	**–0.053**	**0.021**	0.011*	0.52
	Quintile 2 vs. 3	–0.009	0.016	0.58	0.045	0.028	0.11	**–0.047**	**0.019**	**0.017**	0.007
	Quintile 4 vs. 3	–0.012	0.015	0.43	0.006	0.028	0.82	**–0.045**	**0.019**	**0.021**	0.13
	Quintile 5 vs. 3	**–0.041**	**0.016**	0.013*	–0.038	0.03	0.20	**–0.042**	**0.02**	**0.039**	0.91
Temporal	Quintile 1 vs. 3	–0.019	0.018	0.28	–0.041	0.026	0.13	–0.014	0.023	0.55	0.44
	Quintile 2 vs. 3	–0.024	0.016	0.14	–0.008	0.025	0.74	**–0.049**	**0.022**	**0.026**	0.22
	Quintile 4 vs. 3	–0.02	0.016	0.22	–0.06	0.025	0.019	**–0.043**	**0.022**	**0.046**	0.61
	Quintile 5 vs. 3	**–0.054**	**0.017**	0.002*	**–0.062**	**0.027**	**0.026**	–0.043	0.022	0.056	0.59
Occipital	Quintile 1 vs. 3	**–0.037**	**0.017**	0.03*	–0.02	0.028	0.47	**–0.065**	**0.021**	0.003*	0.20
	Quintile 2 vs. 3	–0.016	0.016	0.31	0.021	0.027	0.43	–0.034	0.02	0.094	0.10
	Quintile 4 vs. 3	–0.014	0.015	0.35	–0.004	0.027	0.87	–0.027	0.02	0.17	0.49
	Quintile 5 vs. 3	–0.016	0.016	0.32	–0.018	0.028	0.54	–0.016	0.02	0.44	0.95
Cingulate	Quintile 1 vs. 3	0.02	0.023	0.37	–0.007	0.035	0.83	0.013	0.028	0.65	0.66
	Quintile 2 vs. 3	–0.02	0.02	0.32	0.022	0.034	0.52	**–0.078**	**0.027**	0.004*	0.021
	Quintile 4 vs. 3	–0.004	0.02	0.85	–0.034	0.034	0.31	**–0.056**	**0.026**	**0.034**	0.61
	Quintile 5 vs. 3	**–0.06**	**0.022**	0.006*	–0.056	0.036	0.12	–0.05	0.027	0.068	0.89
Insula	Quintile 1 vs. 3	0.039	0.026	0.14	–0.021	0.039	0.60	0.024	0.034	0.48	0.38
	Quintile 2 vs. 3	0.013	0.024	0.59	–0.003	0.037	0.94	–0.044	0.032	0.17	0.40
	Quintile 4 vs. 3	–0.004	0.024	0.85	–0.095	0.037	0.012	**–0.069**	**0.032**	**0.031**	0.60
	Quintile 5 vs. 3	**–0.059**	**0.025**	0.019*	**–0.112**	**0.04**	**0.006**	–0.052	0.033	0.11	0.25

SE, standard error. Beta coefficients were from generalized linear models, adjusting for age, sex, educational years, hypertension, diabetes, dyslipidemia, angina or myocardial infarction, smoking status, alcohol consumption, apolipoprotein status, body mass index, fasting blood glucose level, total cholesterol level, and intracranial volume. The quintile 3 group was set as the reference group. Significant findings are highlighted in bold. *Significant (p < 0.05) after correction for multiple comparisons, using the false discovery rate method. ^†^Significance of sex differences.

In the GLM analysis of subcutaneous fat (Supplementary Table 1), there were no significant differences in global cortical thickness across the quintile groups. The quintile 4 group had significantly reduced frontal (β = –0.03 mm, *SE* = 0.02 mm, *p* = 0.03), temporal (β = –0.03 mm, *SE* = 0.02 mm, *p* = 0.04), and occipital thicknesses (β = –0.03 mm, *SE* = 0.02 mm, *p* = 0.03), as compared with the quintile 3 group. These associations did not remain significant after FDR correction. After stratification by sex, none of the associations remained significant.

### Association Between Abdominal Fat Area and Subcortical Volume

In the GLM analysis of visceral fat area (Table 4), an increase in visceral fat area was significantly associated with reduced volumes of the pallium (β = –0.66 mm^3^, *SE* = 0.25 mm^3^, *p* = 0.01) and putamen (β = –1.36 mm^3^, *SE* = 0.63 mm^3^, *p* = 0.03). In men, the association between visceral fat area and the reduced volume of the pallidum was significant (β = –0.55 mm^3^, *SE* = 0.25 mm^3^, *p* = 0.03). In women, the association between visceral fat area and the reduced volume of the putamen was significant (β = –1.78 mm^3^, *SE* = 0.90 mm^3^, *p* = 0.05). None of the associations between subcutaneous fat area and subcortical volumes were significant. After FDR correction, none of the associations remained significant.

**TABLE 4:** Association between abdominal fat area and subcortical volume.

	**Total (*N* = 316)**			**Men (*N* = 129)**			**Women (*N* = 187)**			***p* for interaction^†^**
	**Beta**	***SE***	***p***	**Beta**	***SE***	***p***	**Beta**	***SE***	***p***	
**Visceral fat**										
Thalamus	–0.383	0.643	0.55	0.2	0.988	0.84	–0.935	0.913	0.31	0.40
Caudate	–0.928	0.609	0.13	–0.742	0.951	0.44	–1.144	0.877	0.19	0.76
Pallidum	**–0.664**	**0.25**	**0.008**	**–0.79**	**0.348**	**0.025**	–0.584	0.385	0.13	0.69
Putamen	**–1.355**	**0.627**	**0.031**	–1.247	0.983	0.21	**–1.781**	**0.895**	**0.048**	0.69
Amygdala	0.203	0.224	0.37	0.226	0.346	0.52	0.164	0.33	0.62	0.90
Hippocampus	–0.286	0.428	0.50	–0.129	0.626	0.84	–0.597	0.641	0.35	0.60
Nucleus accumbens	–0.132	0.093	0.16	–0.1	0.135	0.46	–0.186	0.136	0.17	0.65
**Subcutaneous fat**										
Thalamus	–0.217	0.624	0.73	–1.258	1.258	0.32	0.351	0.72	0.63	0.27
Caudate	–0.602	0.592	0.31	–1.645	1.209	0.18	–0.445	0.692	0.52	0.39
Pallidum	–0.008	0.246	0.97	–0.026	0.455	0.95	0.051	0.305	0.87	0.89
Putamen	0.307	0.612	0.62	0.877	1.263	0.49	0.134	0.712	0.85	0.61
Amygdala	0.047	0.218	0.83	0.278	0.442	0.53	–0.077	0.26	0.77	0.49
Hippocampus	–0.148	0.416	0.72	0.722	0.797	0.37	–0.395	0.504	0.44	0.24
Nucleus accumbens	–0.157	0.09	0.082	–0.091	0.173	0.60	–0.165	0.107	0.13	0.72

*SE, standard error. Beta coefficients were from generalized linear models, adjusting for age, sex, educational years, hypertension, diabetes, dyslipidemia, angina or myocardial infarction, smoking status, alcohol consumption, apolipoprotein status, body mass index, fasting blood glucose level, total cholesterol level, and intracranial volume. Significant findings are highlighted in bold. None of the associations remained significant after correction for multiple comparisons using the false discovery rate method. ^†^Significance of sex differences.*

### *Post hoc* Analyses

The quintile 1 group of BMI had significantly reduced global (β = –0.03 mm, *SE* = 0.01 mm, *p* = 0.027), parietal (β = –0.06 mm, *SE* = 0.02 mm, *p* < 0.001), temporal (β = –0.05 mm, *SE* = 0.02 mm, *p* = 0.004), and occipital thicknesses (β = –0.04 mm, *SE* = 0.02 mm, *p* = 0.007), as compared with the quintile 3 group. Other findings are presented in Supplementary Table 2. In the analyses of waist circumference, none of the associations were significant except a reduced parietal thickness in the quintile 1 group (β = –0.04 mm, *SE* = 0.02 mm, *p* = 0.015). Other findings are presented in Supplementary Table 3.

## Discussion

The present study is the first to investigate the associations of visceral and subcutaneous fat area with ROI-based cortical thicknesses and subcortical volumes in elderly individuals without dementia. This neuroimaging study involved a relatively large sample size (*n* = 316) and adjusted for a range of covariates including well-known metabolic risk factors, as well as the apolipoprotein ε4 allele—the major genetic risk factor for Alzheimer’s disease. The main finding was that individuals with the highest level of visceral fat area had significantly reduced cortical thicknesses in the global, parietal, temporal, cingulate, and insular lobes, as compared with those with the middle level of visceral fat area. These associations did not significantly differ by sex. By contrast, none of the regional cortical thicknesses significantly differed between individuals with the highest level and those with the middle level of subcutaneous fat area.

In recent decades, there has been debate surrounding the effect of high BMI on dementia risk. The largest cohort study on this topic (of two million individuals) demonstrated a protective effect of higher BMI (Qizilbash et al., 2015), while a meta-analysis of four studies showed a harmful effect of obesity (Pedditzi et al., 2016). Another cohort study of 1.3 million individuals suggested a harmful effect of higher BMI on dementia risk over > 20 years of follow-up, as well as a protective effect of higher BMI over ≤ 20 years of follow-up, possibly due to reverse causation (Kivimaki et al., 2018). Another meta-analysis of 10 prospective cohort studies demonstrated a significant U-shaped association between BMI and dementia risk, indicating that both underweight individuals and overweight individuals are at risk of dementia (Beydoun et al., 2008). This controversial relationship between BMI and dementia and its underlying mechanisms can be, at least in part, scrutinized by using more intricate biomarkers in imaging studies. An MRI analysis of 1,777 cognitively healthy individuals found a significant inverted U-shaped relationship between central obesity (WHR as a proxy) and global cortical thickness (Kim et al., 2015). In line with this, we found a significant inverted U-shaped relationship between visceral fat area on CT and global cortical thickness. It is noteworthy that, when compared with the middle quintile group of visceral fat area, global cortical thinning was significant in the highest quintile group, but not in the lowest quintile group. This highlights a harmful effect of high visceral fat on brain gray matter, as the previous neuroimaging studies have suggested (Debette et al., 2010; Isaac et al., 2011; Widya et al., 2015; Zsido et al., 2019). Taken together, it is possible that high visceral fat leads to cortical thinning and, hence, contribute to the increased risk of dementia in overweight or obese individuals. Further, in concordance with the previous study using voxel-based morphometry (Isaac et al., 2011), the present study demonstrated that the highest level of visceral fat was significantly associated with reduced thicknesses in association cortices (critical for integrating sensory inputs) such as the temporal and parietal lobes. A similar pattern was observed in the cingulate cortex in the present study. These affected brain cortices correspond to the sites that show atrophy in the early stage of mild cognitive impairment (McDonald et al., 2009). Hence, it is reasonable to suggest that individuals with high visceral fat may initially develop preclinical cortical thinning in the temporal, parietal, and cingulate lobes, followed by clinical outcomes such as mild cognitive impairment.

Given possible correlations between visceral fat area and other obesity indices (e.g., BMI and waist circumference), it is possible that the association between visceral fat and cortical thickness was driven by the impact of other obesity indices. In the present study, there was a significant correlation between BMI and visceral fat area (Supplementary Figure 1), and cortical thinning associated with the highest visceral fat group was found significant in the temporal, parietal, cingulate, and insular lobes, after adjusting for a range of covariates including BMI. Notably, a significant decline in cortical thickness was mainly observed in the highest quintile groups of visceral fat area, whereas cortical thinning was significant only in the lowest quintile groups of BMI or waist circumference (Supplementary Tables 2, 3). This suggests that visceral fat area, compared with BMI and waist circumference, might be a better indicator of obesity-induced cortical thinning. In line with this, an analysis of the Framingham Offspring cohort demonstrated that visceral fat was more strongly associated with decreased cerebral volumes compared with BMI, waist circumference, or subcutaneous fat (Debette et al., 2010). Furthermore, the relationship between visceral fat and cortical thinning is supported by animal studies, demonstrating plausible biological mechanisms such as microglial activation, upregulated pro-inflammatory cytokines in the brain, and increased blood-brain barrier permeability via visceral fat inflammation (Shin et al., 2015; Guo et al., 2020). Epidemiological evidence also suggests that visceral fat deposition may induce systemic inflammation and, in turn, cerebral small-vessel disease (e.g., white matter hyperintensities), which is related to reduced cortical thickness (Lampe et al., 2019; Morys et al., 2021).

There are several limitations to be noted. First, we cannot establish temporality between a high level of visceral fat and cortical thinning due to the cross-sectional nature of the study. Longitudinal investigations are warranted to clarify the temporal relationship between high visceral fat area and cortical thinning. Second, our findings might not be generalizable to other ethnic populations. In particular, the inverted U-shaped relationship between visceral fat area and cortical thickness in our samples might not be present in Western populations, though a meta-analysis including 10 prospective studies demonstrated a U-shaped relationship between BMI and risk of dementia (Beydoun et al., 2008). Last, we included dementia-free individuals based on self-reported information, and this approach might not have captured variation in cognitive health (including subthreshold cognitive impairment). Besides, the MMSE may not be sensitive to mild cognitive impairment (Mitchell, 2009; Yim et al., 2021). Future studies are warranted to assess cognitive health with sufficient granularity to elucidate fat-related cortical thinning.

In conclusion, a high level of visceral fat was significantly associated with a reduced global cortical thickness in the brains of elderly individuals without dementia, movement disorders, or stroke. Cortical thinning associated with the highest level of visceral fat area was significant in the parietal, temporal, cingulate, and insular lobes, whereas cortical thinning associated with the highest level of subcutaneous fat area was not significant in any of the studied lobes. These findings support the role of visceral fat in obesity-induced neurodegeneration.

## Data Availability Statement

The original contributions presented in the study are included in the article/Supplementary Material, further inquiries can be directed to the corresponding author/s.

## Ethics Statement

The study was approved by the Institutional Review Board of Gachon University Gil Medical Center (approval No. GDIRB2015-225). The patients/participants provided their written informed consent to participate in this study.

## Author Contributions

JC, CK, and YN designed the study. JC and YN contributed to the data collection and drafted the manuscript. JC analyzed the data. SS, W-RK, and CK provided significant intellectual input and a critical review of the manuscript. All authors approved the final version of the manuscript.

## Conflict of Interest

The authors declare that the research was conducted in the absence of any commercial or financial relationships that could be construed as a potential conflict of interest.

## Footnotes

1 http://surfer.nmr.mgh.harvard.edu/

## References

[B1] Altman D. G. Bland J. M. (2003). Interaction revisited: the difference between two estimates. *BMJ* 326:219. 10.1136/bmj.326.7382.219 12543843 PMC1125071

[B2] Bell J. A. Kivimaki M. Hamer M. (2014). Metabolically healthy obesity and risk of incident type 2 diabetes: a meta-analysis of prospective cohort studies. *Obes. Rev.* 15 504–515. 10.1111/obr.12157 24661566 PMC4309497

[B3] Benjamini Y. Hochberg Y. (1995). Controlling the false discovery rate: a practical and powerful approach to multiple testing. *J. Royal Stat. Soc. Ser. B Methodol.* 57 289–300.

[B4] Beydoun M. A. Beydoun H. A. Wang Y. (2008). Obesity and central obesity as risk factors for incident dementia and its subtypes: a systematic review and meta-analysis. *Obes. Rev.* 9 204–218. 10.1111/j.1467-789X.2008.00473.x 18331422 PMC4887143

[B5] Bogers R. P. Bemelmans W. J. Hoogenveen R. T. Boshuizen H. C. Woodward M. Knekt P. (2007). Association of overweight with increased risk of coronary heart disease partly independent of blood pressure and cholesterol levels: a meta-analysis of 21 cohort studies including more than 300 000 persons. *Arch. Intern. Med.* 167 1720–1728. 10.1001/archinte.167.16.1720 17846390

[B6] Burggren A. C. Zeineh M. M. Ekstrom A. D. Braskie M. N. Thompson P. M. Small G. W. (2008). Reduced cortical thickness in hippocampal subregions among cognitively normal apolipoprotein E e4 carriers. *Neuroimage* 41 1177–1183. 10.1016/j.neuroimage.2008.03.039 18486492 PMC2601686

[B7] Dale A. M. Fischl B. Sereno M. I. (1999). Cortical surface-based analysis. I. Segmentation and surface reconstruction. *Neuroimage* 9 179–194. 10.1006/nimg.1998.0395 9931268

[B8] Debette S. Beiser A. Hoffmann U. Decarli C. O’donnell C. J. Massaro J. M. (2010). Visceral fat is associated with lower brain volume in healthy middle-aged adults. *Ann. Neurol.* 68 136–144. 10.1002/ana.22062 20695006 PMC2933649

[B9] Dekkers I. A. Jansen P. R. Lamb H. J. (2019). Obesity, brain volume, and white matter microstructure at MRI: a cross-sectional UK biobank study. *Radiology* 291 763–771. 10.1148/radiol.2019181012 31012815

[B10] Desikan R. S. Segonne F. Fischl B. Quinn B. T. Dickerson B. C. Blacker D. (2006). An automated labeling system for subdividing the human cerebral cortex on MRI scans into gyral based regions of interest. *Neuroimage* 31 968–980. 10.1016/j.neuroimage.2006.01.021 16530430

[B11] Fischl B. Dale A. M. (2000). Measuring the thickness of the human cerebral cortex from magnetic resonance images. *Proc. Natl. Acad. Sci. U.S.A.* 97 11050–11055. 10.1073/pnas.200033797 10984517 PMC27146

[B12] Fischl B. Salat D. H. Busa E. Albert M. Dieterich M. Haselgrove C. (2002). Whole brain segmentation: automated labeling of neuroanatomical structures in the human brain. *Neuron* 33 341–355. 11832223 10.1016/s0896-6273(02)00569-x

[B13] Fischl B. Salat D. H. Van Der Kouwe A. J. Makris N. Segonne F. Quinn B. T. (2004a). Sequence-independent segmentation of magnetic resonance images. *Neuroimage* 23(Suppl. 1) S69–S84. 10.1016/j.neuroimage.2004.07.016 15501102

[B14] Fischl B. Van Der Kouwe A. Destrieux C. Halgren E. Segonne F. Salat D. H. (2004b). Automatically parcellating the human cerebral cortex. *Cereb Cortex* 14 11–22. 10.1093/cercor/bhg087 14654453

[B15] Fischl B. Sereno M. I. Dale A. M. (1999). Cortical surface-based analysis. II: inflation, flattening, and a surface-based coordinate system. *Neuroimage* 9 195–207. 10.1006/nimg.1998.0396 9931269

[B16] Gunstad J. Paul R. H. Cohen R. A. Tate D. F. Spitznagel M. B. Grieve S. (2008). Relationship between body mass index and brain volume in healthy adults. *Int. J. Neurosci.* 118 1582–1593. 10.1080/00207450701392282 18853335

[B17] Guo D.-H. Yamamoto M. Hernandez C. M. Khodadadi H. Baban B. Stranahan A. M. (2020). Visceral adipose NLRP3 impairs cognition in obesity via IL-1R1 on CX3CR1+ cells. *J. Clin. Investigation* 130 1961–1976. 10.1172/JCI126078 31935195 PMC7108893

[B18] Hamer M. Batty G. D. (2019). Association of body mass index and waist-to-hip ratio with brain structure: UK biobank study. *Neurology* 92 e594–e600. 10.1212/WNL.0000000000006879 30626649 PMC8093082

[B19] Isaac V. Sim S. Zheng H. Zagorodnov V. Tai E. S. Chee M. (2011). Adverse associations between visceral adiposity, brain structure, and cognitive performance in healthy elderly. *Front. Aging Neurosci.* 3:12. 10.3389/fnagi.2011.00012 21949507 PMC3171695

[B20] Jackson S. Thomas R. (eds). (2004). “Computed tomography (CT): clinical applications of CT,” in *Cross-Sectional Imaging Made Easy*, (Oxford: Churchill Livingstone).

[B21] Kim H. J. Kim C. Jeon S. Kang M. Kim Y. J. Lee J. M. (2015). Association of body fat percentage and waist-hip ratio with brain cortical thickness: a study among 1777 cognitively normal subjects. *Alzheimer Dis. Assoc. Disord.* 29 279–286. 10.1097/WAD.0000000000000079 25626634

[B22] Kivimaki M. Luukkonen R. Batty G. D. Ferrie J. E. Pentti J. Nyberg S. T. (2018). Body mass index and risk of dementia: analysis of individual-level data from 1.3 million individuals. *Alzheimers Dement* 14 601–609. 10.1016/j.jalz.2017.09.016 29169013 PMC5948099

[B23] Kurth F. Levitt J. G. Phillips O. R. Luders E. Woods R. P. Mazziotta J. C. (2013). Relationships between gray matter, body mass index, and waist circumference in healthy adults. *Hum. Brain Mapp.* 34 1737–1746. 10.1002/hbm.22021 22419507 PMC6869996

[B24] Lampe L. Zhang R. Beyer F. Huhn S. Kharabian Masouleh S. Preusser S. (2019). Visceral obesity relates to deep white matter hyperintensities via inflammation. *Ann. Neurol.* 85 194–203. 10.1002/ana.25396 30556596 PMC6590485

[B25] McDonald C. R. Mcevoy L. K. Gharapetian L. Fennema-Notestine C. Hagler D. J. Jr. Holland D. (2009). Regional rates of neocortical atrophy from normal aging to early Alzheimer disease. *Neurology* 73 457–465. 10.1212/WNL.0b013e3181b16431 19667321 PMC2727145

[B26] Medic N. Ziauddeen H. Ersche K. D. Farooqi I. S. Bullmore E. T. Nathan P. J. (2016). Increased body mass index is associated with specific regional alterations in brain structure. *Int. J. Obes (Lond)* 40 1177–1182. 10.1038/ijo.2016.42 27089992 PMC4936515

[B27] Mitchell A. J. (2009). A meta-analysis of the accuracy of the mini-mental state examination in the detection of dementia and mild cognitive impairment. *J. Psychiatr. Res.* 43 411–431. 10.1016/j.jpsychires.2008.04.014 18579155

[B28] Morys F. Dadar M. Dagher A. (2021). Association between mid-life obesity, its metabolic consequences, cerebrovascular disease and cognitive decline. *J. Clin. Endocrinol. Metab.* 2:dgab135. 10.1210/clinem/dgab135 33677592 PMC8475210

[B29] Pedditzi E. Peters R. Beckett N. (2016). The risk of overweight/obesity in mid-life and late life for the development of dementia: a systematic review and meta-analysis of longitudinal studies. *Age Ageing* 45 14–21. 10.1093/ageing/afv151 26764391

[B30] Qizilbash N. Gregson J. Johnson M. E. Pearce N. Douglas I. Wing K. (2015). BMI and risk of dementia in two million people over two decades: a retrospective cohort study. *Lancet Diabetes Endocrinol.* 3 431–436. 10.1016/S2213-8587(15)00033-9 25866264

[B31] Raji C. A. Ho A. J. Parikshak N. N. Becker J. T. Lopez O. L. Kuller L. H. (2010). Brain structure and obesity. *Hum. Brain Mapp.* 31 353–364. 10.1002/hbm.20870 19662657 PMC2826530

[B32] Renehan A. G. Tyson M. Egger M. Heller R. F. Zwahlen M. (2008). Body-mass index and incidence of cancer: a systematic review and meta-analysis of prospective observational studies. *Lancet* 371 569–578. 10.1016/S0140-6736(08)60269-X 18280327

[B33] Ritchie S. J. Cox S. R. Shen X. Lombardo M. V. Reus L. M. Alloza C. (2018). Sex differences in the adult human brain: evidence from 5216 UK biobank participants. *Cereb Cortex* 28 2959–2975. 10.1093/cercor/bhy109 29771288 PMC6041980

[B34] Shin J. A. Jeong S. I. Kim M. Yoon J. C. Kim H. S. Park E. M. (2015). Visceral adipose tissue inflammation is associated with age-related brain changes and ischemic brain damage in aged mice. *Brain Behav. Immun.* 50 221–231. 10.1016/j.bbi.2015.07.008 26184082

[B35] Taki Y. Kinomura S. Sato K. Inoue K. Goto R. Okada K. (2008). Relationship between body mass index and gray matter volume in 1,428 healthy individuals. *Obesity (Silver Spring)* 16 119–124. 10.1038/oby.2007.4 18223623

[B36] Thambisetty M. Wan J. Carass A. An Y. Prince J. L. Resnick S. M. (2010). Longitudinal changes in cortical thickness associated with normal aging. *Neuroimage* 52 1215–1223. 10.1016/j.neuroimage.2010.04.258 20441796 PMC2910226

[B37] Widya R. L. Kroft L. J. M. Altmann-Schneider I. Van Den Berg-Huysmans A. A. Van Der Bijl N. De Roos A. (2015). Visceral adipose tissue is associated with microstructural brain tissue damage. *Obesity* 23 1092–1096. 10.1002/oby.21048 25919926

[B38] Yim Y. Lee J. Y. Oh S. W. Chung M. S. Park J. E. Moon Y. (2021). Comparison of automated brain volume measures by neuroquant vs. Freesurfer in patients with mild cognitive impairment: effect of slice thickness. *Yonsei Med. J.* 62 255–261. 10.3349/ymj.2021.62.3.255 33635016 PMC7934099

[B39] Yokum S. Ng J. Stice E. (2012). Relation of regional gray and white matter volumes to current BMI and future increases in BMI: a prospective MRI study. *Int. J. Obes (Lond)* 36 656–664. 10.1038/ijo.2011.175 21894161 PMC3982917

[B40] Zsido R. G. Heinrich M. Slavich G. M. Beyer F. Kharabian Masouleh S. Kratzsch J. (2019). Association of estradiol and visceral fat with structural brain networks and memory performance in adults. *JAMA Netw. Open* 2:e196126. 10.1001/jamanetworkopen.2019.6126 31225892 PMC6593958

