# Supplementary material for: Association Between Visceral Fat and Brain Cortical Thickness in the Elderly: A Neuroimaging Study
Source: Front Aging Neurosci. 2021 Jun 23;13:694629. doi: 10.3389/fnagi.2021.694629 (PMC8261238; doi:10.3389/fnagi.2021.694629)
Supplement: Supplementary file 1 [file Table_1.DOCX]

**SUPPLEMENTARY DATA**

**Title**

Association between visceral fat and brain cortical thickness in the elderly: A neuroimaging study

**Authors**

Jaelim Cho; Seongho Seo; Woo-Ram Kim; Changsoo Kim; Young Noh

**Table of contents**

Table S1. Association between subcutaneous fat area and cortical thickness

Table S2. Association between body mass index and cortical thickness

Table S3. Association between waist circumference and cortical thickness

Figure S1. Body mass index according to quintile of visceral fat area

**Table S1. Association between subcutaneous fat area and cortical thickness**

|  |  | **Total (N=316)** | | | **Men (N=129)** | | | **Women (N=187)** | | | **p for interaction^†^** |
| --- | --- | --- | --- | --- | --- | --- | --- | --- | --- | --- | --- |
|  |  | **Beta** | **SE** | **p** | **Beta** | **SE** | **p** | **Beta** | **SE** | **p** |  |
| **Global** | **Quintile 1 vs. 3** | -0.018 | 0.015 | 0.252 | -0.008 | 0.024 | 0.72 | -0.004 | 0.02 | 0.831 | 0.898 |
|  | **Quintile 2 vs. 3** | -0.019 | 0.014 | 0.179 | 0.006 | 0.022 | 0.804 | -0.002 | 0.018 | 0.919 | 0.778 |
|  | **Quintile 4 vs. 3** | -0.027 | 0.014 | 0.053 | 0.02 | 0.023 | 0.376 | -0.017 | 0.018 | 0.362 | 0.205 |
|  | **Quintile 5 vs. 3** | -0.012 | 0.016 | 0.423 | 0.002 | 0.025 | 0.946 | 0.008 | 0.02 | 0.697 | 0.851 |
| **Frontal** | **Quintile 1 vs. 3** | -0.021 | 0.017 | 0.21 | -0.006 | 0.025 | 0.813 | -0.007 | 0.022 | 0.739 | 0.976 |
|  | **Quintile 2 vs. 3** | -0.03 | 0.015 | 0.051 | -0.007 | 0.023 | 0.773 | 0.007 | 0.02 | 0.724 | 0.646 |
|  | **Quintile 4 vs. 3** | **-0.033** | **0.015** | **0.029** | 0.00 | 0.024 | 0.984 | -0.022 | 0.021 | 0.279 | 0.49 |
|  | **Quintile 5 vs. 3** | -0.03 | 0.017 | 0.076 | 0.005 | 0.027 | 0.864 | 0.005 | 0.023 | 0.84 | 1.00 |
| **Parietal** | **Quintile 1 vs. 3** | -0.011 | 0.017 | 0.535 | -0.01 | 0.028 | 0.737 | -0.035 | 0.021 | 0.088 | 0.475 |
|  | **Quintile 2 vs. 3** | -0.016 | 0.016 | 0.328 | 0.027 | 0.027 | 0.317 | -0.009 | 0.019 | 0.644 | 0.276 |
|  | **Quintile 4 vs. 3** | -0.014 | 0.016 | 0.387 | 0.022 | 0.028 | 0.422 | -0.032 | 0.019 | 0.098 | 0.111 |
|  | **Quintile 5 vs. 3** | -0.019 | 0.018 | 0.286 | -0.016 | 0.03 | 0.61 | 0.005 | 0.021 | 0.8 | 0.566 |
| **Temporal** | **Quintile 1 vs. 3** | -0.033 | 0.018 | 0.061 | -0.009 | 0.026 | 0.719 | -0.009 | 0.023 | 0.697 | 1.00 |
|  | **Quintile 2 vs. 3** | -0.031 | 0.016 | 0.056 | -0.011 | 0.024 | 0.664 | -0.014 | 0.021 | 0.529 | 0.925 |
|  | **Quintile 4 vs. 3** | **-0.033** | **0.016** | **0.037** | 0.025 | 0.025 | 0.324 | -0.008 | 0.022 | 0.722 | 0.322 |
|  | **Quintile 5 vs. 3** | -0.008 | 0.018 | 0.648 | -0.017 | 0.028 | 0.551 | 0.016 | 0.024 | 0.508 | 0.371 |
| **Occipital** | **Quintile 1 vs. 3** | -0.017 | 0.017 | 0.308 | -0.002 | 0.027 | 0.943 | -0.011 | 0.021 | 0.607 | 0.792 |
|  | **Quintile 2 vs. 3** | -0.018 | 0.016 | 0.243 | 0.019 | 0.025 | 0.434 | 0.01 | 0.02 | 0.614 | 0.779 |
|  | **Quintile 4 vs. 3** | **-0.034** | **0.015** | **0.026** | 0.018 | 0.026 | 0.491 | -0.023 | 0.02 | 0.251 | 0.211 |
|  | **Quintile 5 vs. 3** | -0.025 | 0.017 | 0.15 | -0.023 | 0.028 | 0.428 | -0.01 | 0.022 | 0.651 | 0.715 |
| **Cingulate** | **Quintile 1 vs. 3** | -0.026 | 0.023 | 0.252 | -0.029 | 0.034 | 0.385 | 0.006 | 0.029 | 0.84 | 0.434 |
|  | **Quintile 2 vs. 3** | -0.024 | 0.021 | 0.242 | -0.002 | 0.031 | 0.959 | -0.015 | 0.027 | 0.57 | 0.752 |
|  | **Quintile 4 vs. 3** | -0.031 | 0.021 | 0.128 | 0.057 | 0.033 | 0.086 | -0.02 | 0.027 | 0.468 | 0.071 |
|  | **Quintile 5 vs. 3** | -0.005 | 0.023 | 0.835 | 0.043 | 0.036 | 0.234 | 0.006 | 0.03 | 0.829 | 0.43 |
| **Insula** | **Quintile 1 vs. 3** | 0.003 | 0.027 | 0.918 | 0.005 | 0.04 | 0.894 | 0.031 | 0.034 | 0.361 | 0.62 |
|  | **Quintile 2 vs. 3** | 0.006 | 0.024 | 0.821 | 0.006 | 0.037 | 0.875 | 0.009 | 0.032 | 0.767 | 0.951 |
|  | **Quintile 4 vs. 3** | -0.016 | 0.024 | 0.504 | 0.001 | 0.038 | 0.98 | 0.003 | 0.032 | 0.918 | 0.968 |
|  | **Quintile 5 vs. 3** | 0.012 | 0.027 | 0.653 | 0.018 | 0.042 | 0.678 | 0.025 | 0.035 | 0.475 | 0.898 |

*Abbreviations*: SE, standard error.

*Footnotes.* Beta coefficients were from generalized linear models, adjusting for age, sex, educational years, hypertension, diabetes, dyslipidemia, angina or myocardial infarction, smoking status, alcohol consumption, apolipoprotein status, body mass index, fasting blood glucose level, total cholesterol level, and intracranial volume. The quintile 3 group was set as the reference group. Significant findings are highlighted in bold. None of the associations remained significant after correcting for multiple comparisons using the false discovery rate method.

^†^Significance of sex differences

**Table S2. Association between body mass index and cortical thickness**

|  |  | **Total (N=316)** | | | **Men (N=129)** | | | **Women (N=187)** | | |
| --- | --- | --- | --- | --- | --- | --- | --- | --- | --- | --- |
|  |  | **Beta** | **SE** | **p** | **Beta** | **SE** | **p** | **Beta** | **SE** | **p** |
| **Global** | **Quintile 1 vs. 3** | **-0.031** | **0.014** | **0.027** | -0.019 | 0.023 | 0.428 | -0.033 | 0.018 | 0.07 |
|  | **Quintile 2 vs. 3** | 0 | 0.014 | 0.985 | -0.006 | 0.023 | 0.78 | 0.002 | 0.018 | 0.892 |
|  | **Quintile 4 vs. 3** | 0 | 0.013 | 0.985 | 0.007 | 0.022 | 0.738 | 0.001 | 0.018 | 0.945 |
|  | **Quintile 5 vs. 3** | -0.004 | 0.013 | 0.793 | -0.007 | 0.022 | 0.738 | 0.007 | 0.018 | 0.686 |
| **Frontal** | **Quintile 1 vs. 3** | -0.03 | 0.015 | 0.055 | -0.037 | 0.025 | 0.129 | -0.022 | 0.02 | 0.282 |
|  | **Quintile 2 vs. 3** | 0.005 | 0.015 | 0.739 | -0.003 | 0.024 | 0.908 | 0.012 | 0.02 | 0.54 |
|  | **Quintile 4 vs. 3** | 0.004 | 0.015 | 0.764 | -0.005 | 0.023 | 0.839 | 0.016 | 0.02 | 0.414 |
|  | **Quintile 5 vs. 3** | -0.005 | 0.015 | 0.728 | -0.004 | 0.023 | 0.869 | 0.009 | 0.02 | 0.643 |
| **Parietal** | **Quintile 1 vs. 3** | **-0.057** | **0.016** | **<0.001** | -0.044 | 0.028 | 0.122 | **-0.053** | **0.019** | **0.005** |
|  | **Quintile 2 vs. 3** | -0.006 | 0.015 | 0.706 | -0.012 | 0.028 | 0.668 | -0.007 | 0.019 | 0.692 |
|  | **Quintile 4 vs. 3** | -0.012 | 0.015 | 0.413 | -0.01 | 0.027 | 0.701 | -0.005 | 0.019 | 0.784 |
|  | **Quintile 5 vs. 3** | -0.009 | 0.015 | 0.544 | -0.013 | 0.027 | 0.627 | 0.001 | 0.019 | 0.951 |
| **Temporal** | **Quintile 1 vs. 3** | **-0.047** | **0.016** | **0.004** | -0.035 | 0.026 | 0.179 | **-0.057** | **0.021** | **0.007** |
|  | **Quintile 2 vs. 3** | -0.013 | 0.016 | 0.404 | -0.023 | 0.025 | 0.378 | -0.015 | 0.021 | 0.481 |
|  | **Quintile 4 vs. 3** | -0.017 | 0.015 | 0.282 | 0.004 | 0.024 | 0.877 | -0.033 | 0.021 | 0.113 |
|  | **Quintile 5 vs. 3** | -0.018 | 0.015 | 0.241 | -0.021 | 0.024 | 0.402 | -0.019 | 0.021 | 0.366 |
| **Occipital** | **Quintile 1 vs. 3** | **-0.042** | **0.015** | **0.007** | -0.029 | 0.026 | 0.275 | **-0.045** | **0.019** | **0.022** |
|  | **Quintile 2 vs. 3** | -0.014 | 0.015 | 0.353 | -0.018 | 0.026 | 0.493 | -0.009 | 0.019 | 0.623 |
|  | **Quintile 4 vs. 3** | -0.006 | 0.015 | 0.703 | 0.008 | 0.025 | 0.731 | -0.001 | 0.019 | 0.977 |
|  | **Quintile 5 vs. 3** | -0.011 | 0.015 | 0.448 | -0.016 | 0.025 | 0.522 | -0.001 | 0.019 | 0.94 |
| **Cingulate** | **Quintile 1 vs. 3** | -0.009 | 0.021 | 0.648 | 0.032 | 0.034 | 0.352 | -0.012 | 0.026 | 0.654 |
|  | **Quintile 2 vs. 3** | 0.009 | 0.02 | 0.655 | -0.005 | 0.033 | 0.876 | 0.026 | 0.026 | 0.32 |
|  | **Quintile 4 vs. 3** | 0.014 | 0.02 | 0.481 | 0.017 | 0.032 | 0.583 | 0.03 | 0.026 | 0.248 |
|  | **Quintile 5 vs. 3** | 0.002 | 0.02 | 0.9 | -0.011 | 0.032 | 0.732 | 0.039 | 0.026 | 0.135 |
| **Insula** | **Quintile 1 vs. 3** | -0.001 | 0.024 | 0.959 | 0.003 | 0.039 | 0.941 | -0.008 | 0.031 | 0.807 |
|  | **Quintile 2 vs. 3** | 0.02 | 0.024 | 0.39 | 0.022 | 0.038 | 0.571 | 0.008 | 0.031 | 0.807 |
|  | **Quintile 4 vs. 3** | 0.014 | 0.023 | 0.535 | 0.029 | 0.036 | 0.423 | -0.001 | 0.031 | 0.983 |
|  | **Quintile 5 vs. 3** | 0.02 | 0.023 | 0.394 | 0.02 | 0.037 | 0.585 | 0.014 | 0.031 | 0.657 |

*Abbreviations*: SE, standard error.

*Footnotes*. Range of body mass index (kg/m^2^): quintile 1 (18.4–22.0); quintile 2 (22.1–23.8); quintile 3 (23.9–25.3); quintile 4 (25.4–27.1); quintile 5 (27.2–38.6).

Range of body mass index (kg/m^2^) in men: quintile 1 (18.4–22.4); quintile 2 (22.7–24.1); quintile 3 (24.3–25.4); quintile 4 (25.5–27.0); quintile 5 (27.1–30.3).

Range of body mass index (kg/m^2^) in women: quintile 1 (18.4–21.7); quintile 2 (21.8–23.6); quintile 3 (23.7–25.2); quintile 4 (25.3–27.2); quintile 5 (27.3–38.6).

Beta coefficients were from generalized linear models, adjusting for age, sex, educational years, hypertension, diabetes, dyslipidemia, angina or myocardial infarction, smoking status, alcohol consumption, apolipoprotein status, fasting blood glucose level, total cholesterol level, and intracranial volume. The quintile 3 group was set as the reference group. Significant findings are highlighted in bold.

**Table S3. Association between waist circumference and cortical thickness**

|  |  | **Total (N=316)** | | | **Men (N=129)** | | | **Women (N=187)** | | |
| --- | --- | --- | --- | --- | --- | --- | --- | --- | --- | --- |
|  |  | **Beta** | **SE** | **p** | **Beta** | **SE** | **p** | **Beta** | **SE** | **p** |
| **Global** | **Quintile 1 vs. 3** | -0.009 | 0.013 | 0.478 | -0.046 | 0.029 | 0.109 | -0.03 | 0.017 | 0.081 |
|  | **Quintile 2 vs. 3** | 0.002 | 0.013 | 0.863 | -0.033 | 0.026 | 0.205 | 0.001 | 0.019 | 0.978 |
|  | **Quintile 4 vs. 3** | 0.009 | 0.018 | 0.605 | -0.024 | 0.026 | 0.362 | -0.014 | 0.017 | 0.403 |
|  | **Quintile 5 vs. 3** | 0.003 | 0.012 | 0.811 | -0.04 | 0.029 | 0.168 | -0.007 | 0.018 | 0.69 |
| **Frontal** | **Quintile 1 vs. 3** | -0.017 | 0.015 | 0.26 | -0.05 | 0.03 | 0.1 | -0.024 | 0.019 | 0.207 |
|  | **Quintile 2 vs. 3** | -0.014 | 0.015 | 0.349 | -0.025 | 0.027 | 0.353 | -0.009 | 0.021 | 0.686 |
|  | **Quintile 4 vs. 3** | 0.002 | 0.02 | 0.934 | -0.02 | 0.027 | 0.458 | -0.013 | 0.019 | 0.504 |
|  | **Quintile 5 vs. 3** | -0.006 | 0.014 | 0.665 | -0.039 | 0.031 | 0.207 | -0.009 | 0.02 | 0.675 |
| **Parietal** | **Quintile 1 vs. 3** | **-0.037** | **0.015** | **0.015** | **-0.082** | **0.035** | **0.019** | **-0.054** | **0.018** | **0.003** |
|  | **Quintile 2 vs. 3** | -0.009 | 0.015 | 0.551 | -0.043 | 0.031 | 0.171 | -0.023 | 0.02 | 0.252 |
|  | **Quintile 4 vs. 3** | 0.019 | 0.02 | 0.347 | -0.03 | 0.031 | 0.342 | -0.019 | 0.018 | 0.277 |
|  | **Quintile 5 vs. 3** | 0.005 | 0.014 | 0.705 | -0.056 | 0.035 | 0.113 | -0.006 | 0.019 | 0.741 |
| **Temporal** | **Quintile 1 vs. 3** | 0.001 | 0.015 | 0.935 | -0.03 | 0.032 | 0.363 | -0.026 | 0.02 | 0.204 |
|  | **Quintile 2 vs. 3** | 0.015 | 0.015 | 0.335 | -0.025 | 0.029 | 0.401 | 0.006 | 0.022 | 0.786 |
|  | **Quintile 4 vs. 3** | 0.007 | 0.021 | 0.75 | -0.003 | 0.029 | 0.91 | -0.019 | 0.02 | 0.339 |
|  | **Quintile 5 vs. 3** | 0.013 | 0.014 | 0.373 | -0.02 | 0.033 | 0.542 | -0.009 | 0.021 | 0.673 |
| **Occipital** | **Quintile 1 vs. 3** | -0.009 | 0.015 | 0.553 | -0.044 | 0.032 | 0.177 | -0.031 | 0.018 | 0.093 |
|  | **Quintile 2 vs. 3** | 0.008 | 0.015 | 0.581 | -0.04 | 0.029 | 0.173 | 0.006 | 0.02 | 0.751 |
|  | **Quintile 4 vs. 3** | 0.016 | 0.02 | 0.431 | -0.007 | 0.029 | 0.811 | -0.014 | 0.018 | 0.446 |
|  | **Quintile 5 vs. 3** | 0.013 | 0.014 | 0.349 | -0.05 | 0.032 | 0.124 | -0.002 | 0.019 | 0.914 |
| **Cingulate** | **Quintile 1 vs. 3** | -0.004 | 0.02 | 0.824 | -0.044 | 0.041 | 0.29 | -0.045 | 0.025 | 0.075 |
|  | **Quintile 2 vs. 3** | 0.017 | 0.02 | 0.378 | -0.062 | 0.037 | 0.101 | -0.019 | 0.028 | 0.495 |
|  | **Quintile 4 vs. 3** | 0.014 | 0.026 | 0.584 | -0.032 | 0.037 | 0.4 | -0.028 | 0.025 | 0.265 |
|  | **Quintile 5 vs. 3** | 0.007 | 0.018 | 0.687 | -0.075 | 0.042 | 0.073 | -0.015 | 0.026 | 0.569 |
| **Insula** | **Quintile 1 vs. 3** | 0.008 | 0.023 | 0.726 | -0.028 | 0.048 | 0.556 | 0 | 0.029 | 0.998 |
|  | **Quintile 2 vs. 3** | -0.004 | 0.023 | 0.867 | -0.004 | 0.043 | 0.933 | 0.041 | 0.032 | 0.21 |
|  | **Quintile 4 vs. 3** | -0.001 | 0.031 | 0.963 | -0.05 | 0.043 | 0.246 | 0.007 | 0.029 | 0.799 |
|  | **Quintile 5 vs. 3** | -0.015 | 0.021 | 0.497 | -0.001 | 0.048 | 0.984 | -0.002 | 0.031 | 0.941 |

*Abbreviations*: SE, standard error.

*Footnotes*. Range of waist circumference (cm): quintile 1 (61.0–73.7); quintile 2 (74.9–77.5); quintile 3 (78.7–81.3); quintile 4 (82.6–85.1); quintile 5 (86.4–101.6).

Range of waist circumference (cm) in men: quintile 1 (71.1–77.5); quintile 2 (78.7–81.3); quintile 3 (82.6–85.1); quintile 4 (86.4–87.6); quintile 5 (88.9–101.6).

Range of waist circumference (cm) in women: quintile 1 (61.0–71.1); quintile 2 (72.4–73.7); quintile 3 (76.2–78.7); quintile 4 (80.0–81.3); quintile 5 (82.6–97.8).

Beta coefficients were from generalized linear models, adjusting for age, sex, educational years, hypertension, diabetes, dyslipidemia, angina or myocardial infarction, smoking status, alcohol consumption, apolipoprotein status, fasting blood glucose level, total cholesterol level, and intracranial volume. The quintile 3 group was set as the reference group. Significant findings are highlighted in bold.


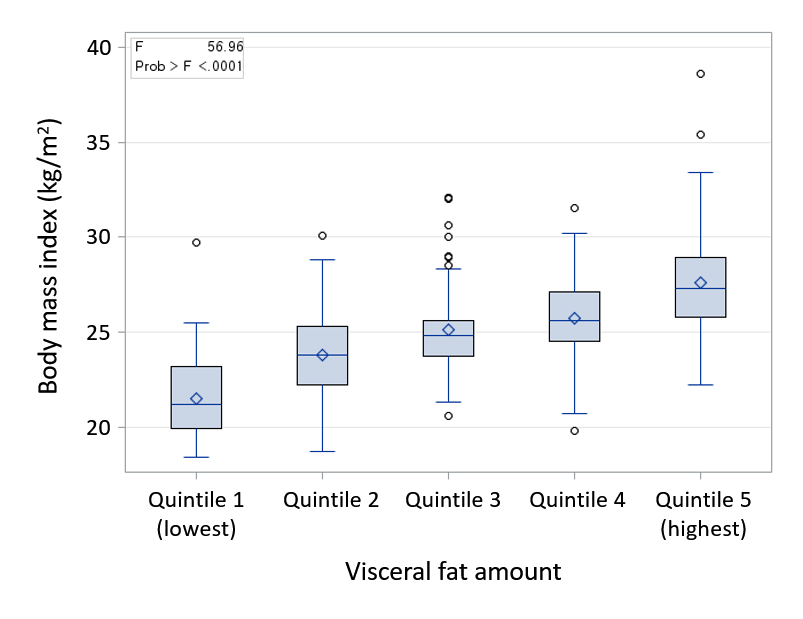


**Figure S1. Body mass index according to quintile of visceral fat area**

*Footnotes*. F-values and P-values were derived from Analysis of Variances. The length of the box indicates the interquartile range. In each box interior, diamonds indicate mean values and horizontal lines represent the group median. The vertical lines emerging from the box extend to the minimum and maximum values.
